# Conditional random field approach to prediction of protein-protein interactions using domain information

**DOI:** 10.1186/1752-0509-5-S1-S8

**Published:** 2011-06-20

**Authors:** Morihiro Hayashida, Mayumi Kamada, Jiangning Song, Tatsuya Akutsu

**Affiliations:** 1 Bioinformatics Center, Institute for Chemical Research, Kyoto University, Gokasho, Uji, Kyoto, 611-0011, Japan; 2 Department of Biochemistry and Molecular Biology, Monash University, Clayton, VIC 3800, Australia; 3 Tianjin Institute of Industrial Biotechnology, Chinese Academy of Sciences, Tianjin 300308, China

## Abstract

**Background:**

For understanding cellular systems and biological networks, it is important to analyze functions and interactions of proteins and domains. Many methods for predicting protein-protein interactions have been developed. It is known that mutual information between residues at interacting sites can be higher than that at non-interacting sites. It is based on the thought that amino acid residues at interacting sites have coevolved with those at the corresponding residues in the partner proteins. Several studies have shown that such mutual information is useful for identifying contact residues in interacting proteins.

**Results:**

We propose novel methods using conditional random fields for predicting protein-protein interactions. We focus on the mutual information between residues, and combine it with conditional random fields. In the methods, protein-protein interactions are modeled using domain-domain interactions. We perform computational experiments using protein-protein interaction datasets for several organisms, and calculate AUC (Area Under ROC Curve) score. The results suggest that our proposed methods with and without mutual information outperform EM (Expectation Maximization) method proposed by Deng et al., which is one of the best predictors based on domain-domain interactions.

**Conclusions:**

We propose novel methods using conditional random fields with and without mutual information between domains. Our methods based on domain-domain interactions are useful for predicting protein-protein interactions.

## Background

Understanding of protein functions and protein-protein interactions is one of important topics in the field of molecular biology and bioinformatics. Recently, many researchers have focused on the investigation of amino acid residues of proteins to reveal interactions and contacts between residues [1-4]. If residues at important sites for interactions between proteins are substituted in one protein, the corresponding residues in interacting partner proteins are expected to be also substituted by selection pressure. Otherwise, such mutated proteins may lose the interactions. Fraser et al. confirmed that interacting proteins evolve at similar evolutionary rates by comparing putatively orthologous protein sequences between *S. cerevisiae* and *C. elegans*[5]. It means that substitutions for contact residues occur in both interacting proteins as long as the proteins keep interacting with each other. Therefore, mutual information (MI) between residues is useful for predicting protein-protein interactions for proteins of unknown function. MI is calculated from multiple sequence alignments for homologous protein sequences. Weigt et al. identified direct residue contacts between sensor kinase and response regulator proteins by message passing, which is an improvement of MI [4]. Burger and van Nimwegen used a dependence tree where a node corresponds to a position of amino acid sequences, and predicted interactions using a Bayesian network method [2]. On the other hand, Markov random field and conditional random field models have been well studied in fields of natural language processing [6,7]. Also in bioinformatics, protein function prediction methods from protein-protein interaction network and other biological networks were developed using Markov random fields [8,9]. On the other hand, several prediction methods have been developed based on domain-domain interactions. Deng et al. proposed a domain-based probabilistic model of protein-protein interactions, and developed EM (Expectation Maximization) method [10]. Based on this probabilistic model, LP (Linear Programming)-based methods were developed [11], and Chen et al. improved the accuracy of interaction strength prediction by APM (Association Probabilistic Method) [12]. In this paper, we propose prediction methods based on domain-domain interactions using conditional random fields with and without mutual information. Furthermore, we perform computational experiments for several protein-protein interaction datasets, compare the methods with the EM method proposed by Deng et al. [10], which is one of the best predictors based on domain-domain interactions, and the association method proposed by Sprinzak and Margalit [13] (the APM method for binary interaction data is equivalent to the association method), and show that our methods outperform the EM method and the association method.

## Mutual information between domains

In order to investigate the relationship between two positions of proteins, MI for distributions of amino acids at the positions is used. Such distributions can be obtained from multiple alignments of protein sequences and domain sequences. In this section, we briefly review MI for distributions of amino acids, and explain MI between domains.

**Figure 1:**
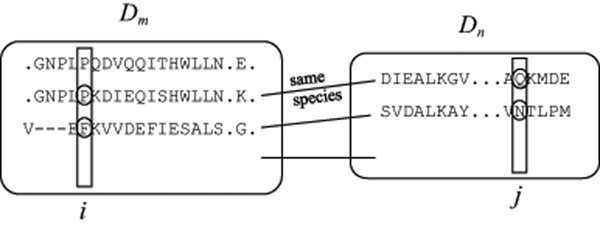
**Illustration on the calculation of mutual information from multiple alignments of domains** Domains *D_m_* and *D_n_* have multiple alignments of sequences from several organisms, respectively. Mutual information is calculated for each pair of positions *i* and *j*.

We assume that multiple sequence alignments for domains *D_m_* and *D_n_* are obtained, respectively (see Figure 1). In order to calculate MI, we need joint appearance frequencies. However, we cannot see which sequence in the multiple alignment of domain *D_m_* corresponds to a specified sequence in that of *D_n_*. Therefore, we assume that sequences contained in the same organism can be paired. In the example of Figure 1, the second sequence of *D_m_* is paired with the first one of *D_n_*, the third one of *D_m_* is paired with the second one of *D_n_*, and so on. The first sequence of *D_m_* is not counted into the appearance frequencies because it is not paired with any sequence of *D_n_* although it may be paired with sequences of other domains than *D_n_*.

Let ***A*** be a set of amino acids, *f_i_*(*A*) be the appearance frequency of amino acid *A* at position *i* in domains *D_m_* and *D_n_*, and *f_ij_*(*A*, *B*) be the joint appearance frequency of a pair of amino acids *A* at position *i* in *D_m_* and *B* at position *j* in *D_n_*, where each frequency is divided by the number of paired sequences *M* in the multiple alignments such that ∑_*A*∈***A***_*f_i_*(*A*) = ∑_*A*,*B*∈***A***_*f_ij_*(*A*,*B*) = 1.

Multiple alignments often include some gaps. Weigt et al. counted the frequencies of gaps as well as amino acids [4]. Therefore, we also consider gaps to be a kind of amino acids, that is, the number of distinct amino acids is |***A***| = 21. Then, mutual information for positions *i* in *D_m_* and *j* in *D_n_* is defined as the Kullback-Leibler divergence between the multiplication of appearance frequencies, *f_i_*(*A*)*f_j_*(*B*), and the joint appearance frequencies, *f_ij_*(*A*,*B*), as follows.(1)

If frequency distributions of amino acids at positions *i* and *j* are independent from each other, *f_ij_*(*A*,*B*) ≈ *f_i_*(*A*)*f_j_*(*B*), and *MI_ij_* approaches to zero. This means that the two positions are not related with each other in the evolutionary process. If domains *D_m_* and *D_n_* interact at the positions, it is considered that *MI_ij_* becomes high because the positions have coevolved through the evolutionary process in order to keep the interaction. It should be noted that two positions *i* and *j* do not always directly interact even if *MI_ij_* is high [4]. However, such proteins with high values of MI have a possibility to directly interact with each other at other positions in the proteins.

However, we need to reduce *MI_ij_* because it can be unnecessarily high depending on distributions of *f_i_*(*A*) and *f_j_*(*B*). For that purpose, we make use of , which is the mutual information *MI_ij_* from the joint frequency, *f_ij_*(*A*, *B*), obtained by shuffling at random the combinations of sequences in multiple alignments. In this paper, we repeat the procedure 400 times according to [4], and take the average. For practical uses of MI, *f_i_*(*A*), *f_j_*(*B*) and *f_ij_*(*A*,*B*) should be positive values. Otherwise, we cannot calculate *MI_ij_* by using computers. Therefore, we use the following pseudocount as in [4],(2)(3)

where *η* is a constant value, in this paper we use *η* = 1. It should be noted that the sum over all amino acids ***A***,  and  because ∑_*A*∈***A***_*f_i_*(*A*) = ∑_*A*,*B*∈***A***_*f_ij_*(*A*,*B*) = 1.

In order to investigate interactions between proteins, we need MI between domains included in the proteins. Thus, we define MI between domains *D_m_* and *D_n_*, ***M****_mn_*, to be the maximum of MI over all positions *i* and *j* as follows.(4)

where 〈*v*〉 means the average of *v*, *i* and *j* are positions of *D_m_* and *D_n_*, respectively. Since *MI_ij_* is calculated to be high for the positions *i* and *j* that include many gaps, we exclude positions that include more than 20% gaps as in [14].

## Conditional random field model for PPI

**Figure 2:**
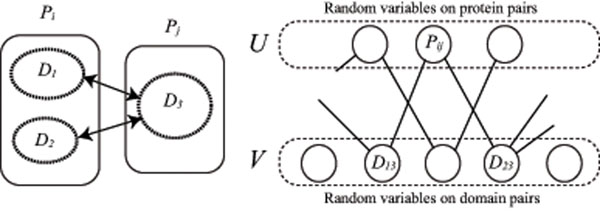
**Markov random field model for protein-protein interactions** Left: Example of proteins *P_i_* and *P_j_*. *P_i_* consists of domains *D*_1_ and *D*_2_, and *P_j_* consists of domain *D*_3_, respectively. Right: Factor graph *G*(*U*,*V*,*E*). There exists an edge between *P_ij_* ∈ *U* and *D_mn_* ∈ *V* if and only if *D_mn_* ∈ *P_ij_*.

In this section, we propose a probabilistic model for protein-protein and domain-domain interactions using conditional random fields [6,7] because it can be considered that two domains *D_m_* and *D_n_* do not always interact even if the mutual information ***M****_mn_* is large. For example, Weigt et al. improved MI and proposed direct information (DI) because residues do not always contact with each other even if the MI is large [4]. Most proteins contain domains as is well known. If two proteins do not interact with each other, any two domains contained in the proteins must not interact with each other. In the left example of Figure 2, protein *P_i_* consists of domains *D*_1_ and *D*_2_ and protein *P_j_* consists of domain *D*_3_ respectively. If *P_i_* and *P_j_* do not interact, any pair of (*D*_1_, *D*_3_) and (*D*_3_, *D*_3_) does not interact. Deng et al. proposed a probabilistic model for a pair of proteins as follows [10]. By assuming that proteins *P_i_* and *P_j_* interact if and only if at least a pair of domains included in the proteins interacts, and events that domains interact are independent from each other, they defined(5)

where *P_ij_* = 1 means that proteins *P_i_* and *P_j_* interact, *D_mn_* = 1 means that domains *D_m_* and *D_n_* interact, *D_mn_* ∈ *P_ij_* means that domain *D_m_* is included in protein *P_i_* and *D_n_* is included in *P_j_* and the product in the right hand side is calculated for all domain pairs (*D_m_*, *D_n_*) included in the protein pair (*P_i_*, *P_j_*). By transforming equation (5), we have(6)(7)

where *λ*^(*mn*)^ = log(1 – *Pr*(*D_mn_* = 1)).

From this equation, we can consider the following Markov random field model for protein pair (*P_i_*, *P_j_*) (see Figure 2).(8)

where *p_ij_* ∈ {0, 1}, ***d*** means a set of events on domain-domain interactions, *D_mn_* = *d_mn_* (*d_mn_* ∈ {0, 1}),  denotes a local feature,  is the corresponding weight parameter and related to the joint probability *Pr*(*P_ij_* = *s*, *D_mn_* = *t*), and Z*_ij_* denotes the normalization constant. For instance, equation (8) for *p_ij_* = 0 is equivalent to equation (7) in the case that  for all protein pairs (*P_i_*, *P_j_*) and  if *s* = *t* = 0, otherwise 0.

In Markov random fields, random variables have Markov properties represented as an undirected graph [15]. The factor graph for our model is represented to be a bipartite graph *G*(*U*, *V*, *E*) with a set of vertices *U* corresponding to protein-protein interactions *P_ij_*, a set of vertices *V* corresponding to domain-domain interactions *D_mn_*, and a set of edges *E* between *U* and *V* as the right figure of Figure 2. There exists an edge between *P_ij_* ∈ *U* and *D_mn_* ∈ *V* if and only if *D_mn_* ∈ *P_ij_*. For the left example of Figure 2, protein pair (*P_i_*, *P_j_*) includes domain pairs (*D*_1_, *D*_3_) and (*D*_2_, *D*_3_). Then, in the factor graph, the vertex of *P_ij_* is connected with vertices of *D*_13_ and *D*_23_, respectively. Although the vertex of *P_ij_* does not have other adjacent vertices than the vertices of *D*_13_ and *D*_23_, those of *D*_13_ and *D*_23_ can be connected with other vertices than that of *P_ij_*

Since *Pr*(*P_ij_* = 0|*D_mn_* =*t*) = 1 – *Pr*(*P_ij_* = 1|*D_mn_* = *t*), it is redundant to consider both *s* = 0, 1, and it is sufficient to consider only *s* = 1. Therefore, in order to simplify the model, we substitute , , and  for all protein pairs (*P_i_*, *P_j_*). Then, we have the following joint probability,(9)

where ***p*** means a set of events on protein-protein interactions, *P_ij_* = *p_ij_*.

We here introduce mutual information between domains ***M*** = {***M****_mn_*} as given conditional data in order to combine it with the probabilistic model. Then, equation (9) can be written as(10)

where(11)(12)

*σ*(*x*) = 1/(1 + *e*^–*x*^) is an increasing function, and *c* is a positive constant. It should be noted that a negative value, –1, is given to  because it is undesired that a pair of domains interact although proteins having the pair do not interact. In this way, the local feature  correlates protein-protein interactions *P_ij_* with domain-domain interactions *D_mn_* (see Figure 2).

For a conditional random field model without MI, we use the following local feature instead of .(13)

### Parameter estimation

In this section, we discuss how to estimate the parameters . We assume that protein-protein interaction data ***p*** = {*p_ij_*} are given. Then, the likelihood function is represented by(14)

where *Z*(***M***) = ∏_*p_ij_*∈***p***_*Z_ij_*(***M***). By taking the logarithm, we have(15)

We estimate the parameters by maximizing the log-likelihood function, *l*(***λ***). Since log(*e^x^* + *e^y^*) is a convex function for variables *x* and *y*, that is, *l*(***λ***) is a concave function, we are able to obtain a global maximum. For maximizing such functions, various methods such as the steepest descent method, Newton’s method, and the Broyden-Fletcher-Goldfarb-Shanno (BFGS) [16] method have been developed. Newton’s method calculates the inverse of the Hessian matrix for the objective function. However, the computational cost is high. Therefore, the quasi-Newton method approximates the matrix by some efficient method using the first derivatives, the gradient. In this paper, we use the BFGS method, which is one of the quasi-Newton methods. By differentiating equation (15) partially with respect to each parameter , we have(16)

In the BFGS method, this equation is repeatedly applied for updating a solution.

## Computational experiments

### Data and implementation

We used protein-protein interaction data of *H. sapiens*, *D. melanogaster*, and *C. elegans* from the DIP database [17], the file name is ’dip20091230.txt’. We used the UniProt Knowledgebase database (version 15.4) [18] as protein domain inclusion data. We deleted proteins that did not have any domain, and obtained 294 interacting protein pairs as positive data that included 300 distinct proteins and 320 domains for *H. sapiens*, 449 interacting pairs that included 562 proteins and 449 domains for *D. melanogaster*, and 250 interacting pairs that included 602 proteins and 476 domains for *C. elegans*.

**Figure 3:**
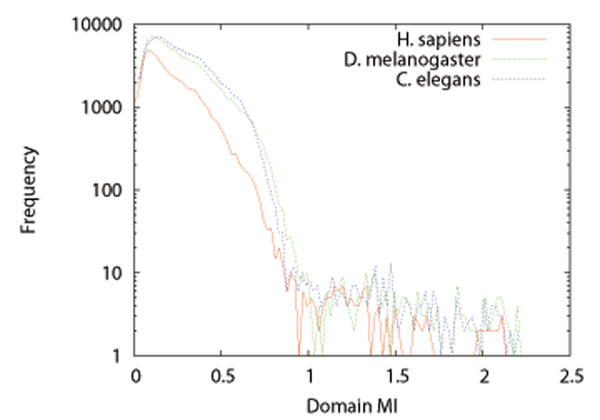
Distributions of domain MIs for *H. sapiens*, *D. melanogaster*, and *C. elegans*

We used the Pfam database (version 24.0) [19] to obtain multiple sequence alignments for domains, and calculated MI, ***M****_mn_*, for each pair of domains. Figure 3 shows the distributions of domain MI ***M****_mn_* for *H. sapiens*, *D. melanogaster*, and *C. elegans*. We can see from the figure that most domain MIs are distributed in the part of less than about 0.8 for all organisms. It is considered that domains *D_m_* and *D_n_* with ***M****_mn_* less than 0.8 may not interact, and domains with ***M****_mn_* more than 0.8 have more possibilities to interact with each other. Therefore, we set the constant *c* in equation (12) to be 0.8. Although we tried several values from 0.6 to 1.0 for *c*, the results were similar to the case of *c* = 0.8.

We selected non-interacting protein pairs as negative data uniformly at random such that negative data did not overlap with the positive data. The number of negative data was the same as that of positive data for each organism.

We used libLBFGS (version 1.9) with default parameters to estimate the parameters , which is a C implementation of the limited memory BFGS method [20], and is available on the web page, http://www.chokkan.org/software/liblbfgs/.

### Result

In order to evaluate our method, we compared the proposed CRF method with MI and that without MI with the EM method by Deng et al. [10] and the association method proposed by Sprinzak and Margalit [13]. The association method and the APM method [12] estimate probabilities *λ_mn_* that domains *D_m_* and *D_n_* interact as  and , respectively, where *N_mn_* (*I_mn_*) denotes the number of (interacting) protein pairs that include domain pair (*D_m_*, *D_n_*), and *ρ_ij_* denotes the interaction strength of protein pair (*P_i_*, *P_j_*), 0 ≤ *ρ_ij_* ≤ 1. However, our input interaction data are binary, that is, *ρ_ij_* takes only 0 or 1. Then, the numerator of the APM method becomes *I_mn_*. It means that the APM method for binary interaction data is equivalent to the association method. In the EM method, probabilities *λ_mn_* that domains *D_m_* and *D_n_* interact are estimated by the recursive formula, , where *o_ij_* = 1 denotes that it was observed that proteins *P_i_* and *P_j_* interact with each other, and *fn* = 0.8. In this paper, the solution of the association method was given as the initial value  of the EM method.

**Figure 4:**
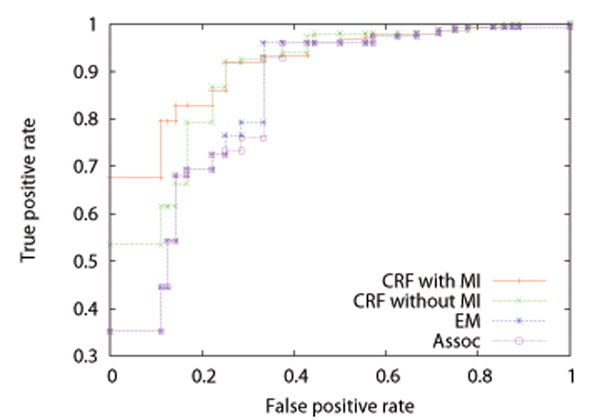
Average ROC curves for test datasets of *H. sapiens* by the CRF method with MI, that without MI, the EM method, and the association method

**Figure 5:**
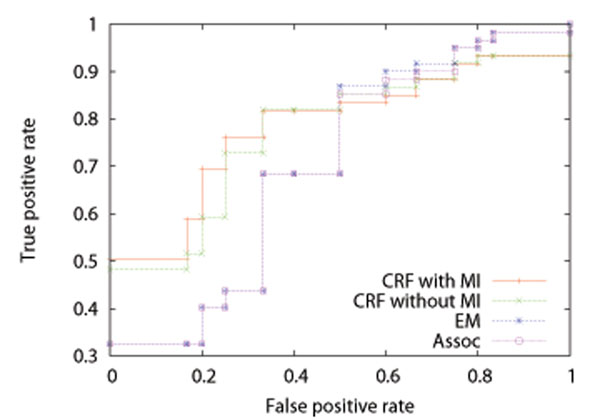
Average ROC curves for test datasets of *D. melanogaster* by the CRF method with MI, that without MI, the EM method, and the association method

**Figure 6:**
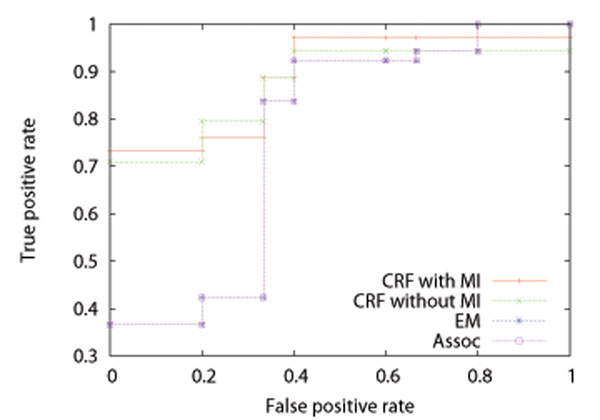
Average ROC curves for test datasets of *C. elegans* by the CRF method with MI, that without MI, the EM method, and the association method

We performed five-fold cross-validation, that is, split the data into 5 datasets (4 for training and 1 for test), estimated  from the training datasets, and calculated *Pr*(*P_ij_* = 1|***M***) of equation (10) for each protein pair in the test dataset and AUC (Area Under ROC Curve) score, where among the test dataset only protein pairs that included at least a parameter estimated from the corresponding training dataset were always used. We repeated 5 times, and took the average. Tables 1, 2, and 3 show the results on AUC for training and test datasets by the CRF method with MI, that without MI, the EM method, and the association method for *H. sapiens*, *D. melanogaster*, and *C. elegans*, respectively. An AUC score is the area under an ROC (Receiver Operating Characteristic) curve, and takes a value between 0 and 1. The ROC curve of a random classifier lies on the diagonal line, and the AUC score is 0.5. The ROC curve of a perfect classifier goes through the point (0 (false positive rate), 1 (true positive rate)), and the AUC score is 1. A classifier with the AUC score closer to 1 has better performance. We can see from these tables that the results by the CRF method with MI are better than those by the CRF method without MI, and that the results by the CRF method without MI are better than those by the EM method and the association method. It is also seen that the results by the EM method are almost the same as those by the association method. It might be because the parameters of the EM method were estimated from the solution of the association method and the solution of the EM method already reached a local optimum. Figures 4, 5, and 6 show the average ROC curves for training and test datasets by the CRF method with MI, that without MI, the EM method, and the association method. For training datasets, the results by all of the methods were almost perfect. For test datasets, the CRF method with MI outperformed that without MI, the EM method, and the association method. It should be noted that the ROC curves of the EM method are almost the same as those of the association method for the same reason discussed above.

**Table 1:** The AUC results for training and test datasets of *H. sapiens* by the CRF method with MI, that without MI, the EM method, and the association method

iteration	CRF with MI		CRF without MI		EM		Assoc	
	
	training	test	training	test	training	test	training	test
1st	0.999366	0.989247	0.999366	0.881720	0.999819	0.709677	0.999602	0.709677

2nd	0.998787	0.919355	0.999312	0.923387	0.999909	0.875000	0.999330	0.854839

3rd	1.000000	0.847222	1.000000	0.833333	1.000000	0.861111	1.000000	0.861111

4th	0.999351	0.989583	0.999369	1.000000	0.999856	0.989583	0.999351	0.989583

5th	0.999333	0.842365	0.999369	0.827586	0.999982	0.798030	0.999802	0.798030

average	0.999367	0.917554	0.999483	0.893205	0.999913	0.846680	0.999617	0.842648

**Table 2:** The AUC results for training and test datasets of *D. melanogaster* by the CRF method with MI, that without MI, the EM method, and the association method

iteration	CRF with MI		CRF without MI		EM		Assoc	
	
	training	test	training	test	training	test	training	test
1st	0.999255	0.707692	0.999977	0.738462	0.999961	0.769231	0.999938	0.769231

2nd	0.997928	0.818182	0.997905	0.848485	0.999938	0.727273	0.999736	0.727273

3rd	0.997920	0.708333	0.997920	0.562500	0.999922	0.645833	0.999884	0.625000

4th	0.998660	0.863636	0.999318	0.886364	0.999814	0.840909	0.999853	0.840909

5th	0.999234	0.819444	0.999954	0.833333	0.999861	0.527778	0.999923	0.527778

average	0.998599	0.783458	0.999015	0.773829	0.999899	0.702205	0.999867	0.698038

**Table 3:** The AUC results for training and test datasets of *C. elegans* by the CRF method with MI, that without MI, the EM method, and the association method

iteration	CRF with MI		CRF without MI		EM		Assoc	
	training	test	training	test	training	test	training	test

1st	0.999975	0.657143	0.999975	0.514286	1.000000	0.542857	1.000000	0.542857

2nd	0.997899	0.923077	0.996873	0.948718	0.999875	0.743590	0.999825	0.743590

3rd	0.998775	0.900000	0.998825	0.933333	0.999875	0.866667	0.999825	0.866667

4th	0.998950	0.966667	0.999850	0.966667	0.999850	0.633333	0.999850	0.633333

5th	0.998900	1.000000	0.998875	1.000000	0.999675	1.000000	0.999700	1.000000

average	0.998900	0.889377	0.998879	0.872601	0.999855	0.757289	0.999840	0.757289

## Conclusions

We proposed novel methods which combine conditional random fields with the domain-based model of protein-protein interactions. In order to give better performance, we introduced mutual information to the probabilistic model. In the improved model, mutual information between domains is given as conditions, where MI between domains is defined as the maximum of MIs between residues in the domains. This method was developed based on the fact that amino acid residues at important sites for interactions have coevolved with each other, and MI has been used for identifying contact residues in interactions. We performed five-fold cross-validation experiments, and calculated AUC for probabilities that two proteins interact. The results suggested that our proposed methods, especially the CRF method with mutual information, are useful. However, the results of AUC for training datasets implied that estimated parameters were overfitting to training datasets. For avoiding that problem, we can improve the methods, for instance, by adding regularization terms, *l*_1_-norm of parameters to the log-likelihood function. Since CRF has an advantage to be able to incorporate large number of features, it remains as a future work to improve the model itself to obtain better accuracy by, for instance, modifying the local feature and adding new features.

## Authors contributions

JS proposed the use of mutual information for predicting protein-protein interactions. Methods were developed and implemented by MH. MK and TA participated in the discussion during development of the methods. The manuscript was prepared by MH, JS, and TA.

## Competing interests

The authors declare that they have no competing interests.
